# Musculoskeletal Tuberculosis of the Pectoralis With Bilateral Pleural Base Nodular Lesions: A Rare Report of Its Type in an Immunocompetent Female

**DOI:** 10.7759/cureus.51804

**Published:** 2024-01-07

**Authors:** Sankalp Yadav

**Affiliations:** 1 Medicine, Shri Madan Lal Khurana Chest Clinic, New Delhi, IND

**Keywords:** mycobacterium tuberculosis (mtb), muscle tuberculosis, culture, cartridge-based nucleic acid amplification test (cbnaat), pectoralis muscle

## Abstract

Musculoskeletal tuberculosis is a rare infection caused by *Mycobacterium tuberculosis*. This type of extrapulmonary tuberculosis is mainly attributed to hematogenous infection or direct inoculation and is usually seen in immunocompromised individuals. Here, a case of musculoskeletal tuberculosis of the right pectoralis with bilateral pleural base nodular lesions in an immunocompetent female is presented. A challenging diagnosis was achieved through a detailed clinical examination, aspiration of the pus, and radiometric investigations. Management was done through antituberculous chemotherapy per the national guidelines. Further, a detailed literature review revealed that tuberculosis of the pectoralis with bilateral pleural base nodular lesions is never reported in an immunocompetent female.

## Introduction

*Mycobacterium tuberculosis* infection is commonly manifested clinically as pulmonary tuberculosis, but extrapulmonary tuberculosis is also reported [1]. However, it is relatively rare, with a prevalence of 8.4-13.7% [2]. Further, only 1-3% of tuberculosis patients develop musculoskeletal tuberculosis, and only 1-5% of these instances involve tuberculosis of the chest wall [3]. Direct inoculation or hematogenous spread from a primary target, such as the lung, may lead to tuberculosis of the tendon and muscles [4].

The musculoskeletal system's primary tuberculosis was initially documented in 1886 and has been noted as rare ever since. Pectoralis major tuberculosis is exceedingly uncommon, with few case reports available [5]. Although the precise cause of muscle tissue's resistance to tubercle bacillus is unknown, the presence of lactic acid, scarcity of reticulo-epithelial tissue, and low oxygen levels in muscle tissue have been suggested [6-7].

A case of 26-year-old Indian immunocompetent female is presented who came with complaints of pain and swelling in her anterior chest wall for three months. A diagnostic workup backed by an extensive clinical examination and radiometric tests helped finalize the diagnosis.

## Case presentation

A 26-year-old Indian non-diabetic, immunocompetent female presented with complaints of pain and swelling in her right upper anterior chest wall for three months. The swelling was insidious in onset and increased to the present size in the last 15 days. It was associated with pain, which was continuous and not associated with any aggravating or relieving factors. However, it subsided for a few hours after she took an over-the-counter analgesic (diclofenac). There was no history of fever, loss of appetite, night sweats, or weight loss. Besides, there was no history of trauma or immunosuppression. And there was no history of any other major medical or surgical intervention, including tuberculosis.

She was a housewife with no history of smoking, alcoholism, or any other substance abuse. Additionally, there was no history of imprisonment or stays at either night shelters or refugee camps.

A general examination revealed a lean female with a temperature of 98.4 degrees Fahrenheit, a respiratory rate of 17/minute, a blood pressure of 120/80 mmHg, and an oxygen saturation of 98% on room air. There was no lymphadenopathy, cyanosis, icterus, pallor, edema, clubbing, or koilonychia present. Her systemic examination was unremarkable.

**Figure 1:**
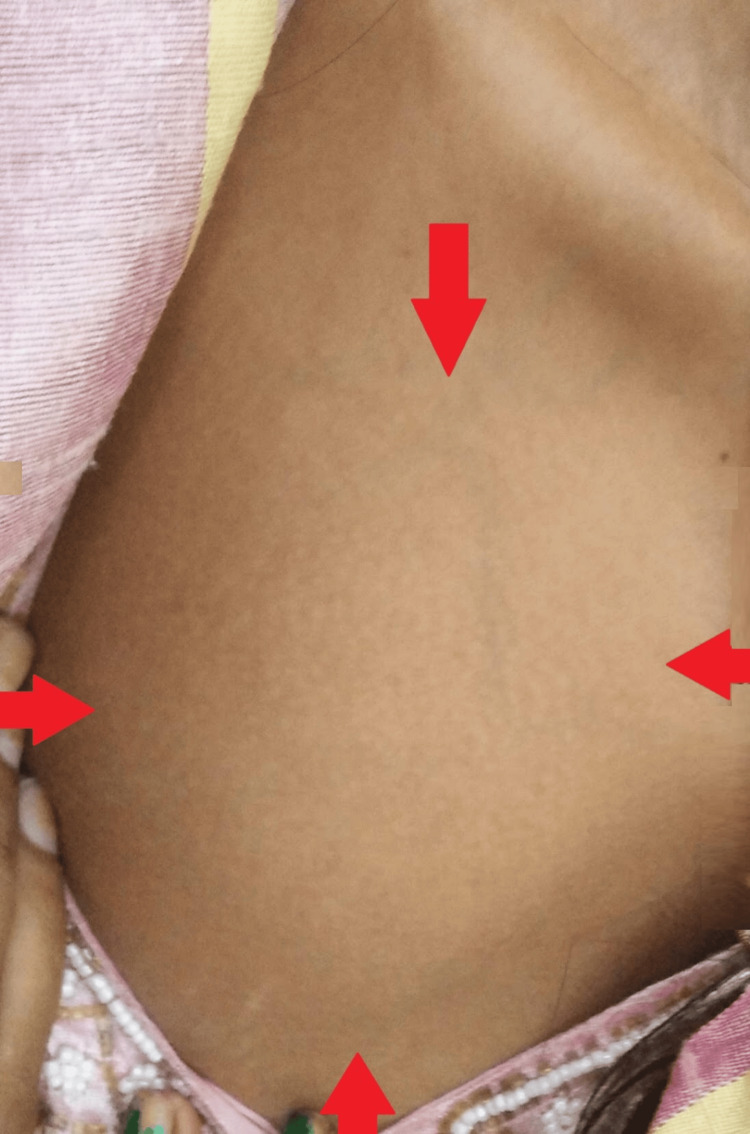
A gross image showing a large infraclavicular swelling

Local examination revealed a large fixed swelling about 4 cm from the right clavicle in the right hemithorax, with the size of 7x5 cm firm on consistency, irregular with a well-defined border, fluctuant, tender to touch, and without discharging sinus. The overlying skin was normal, without any engorged veins (Figure 1).

**Figure 2:**
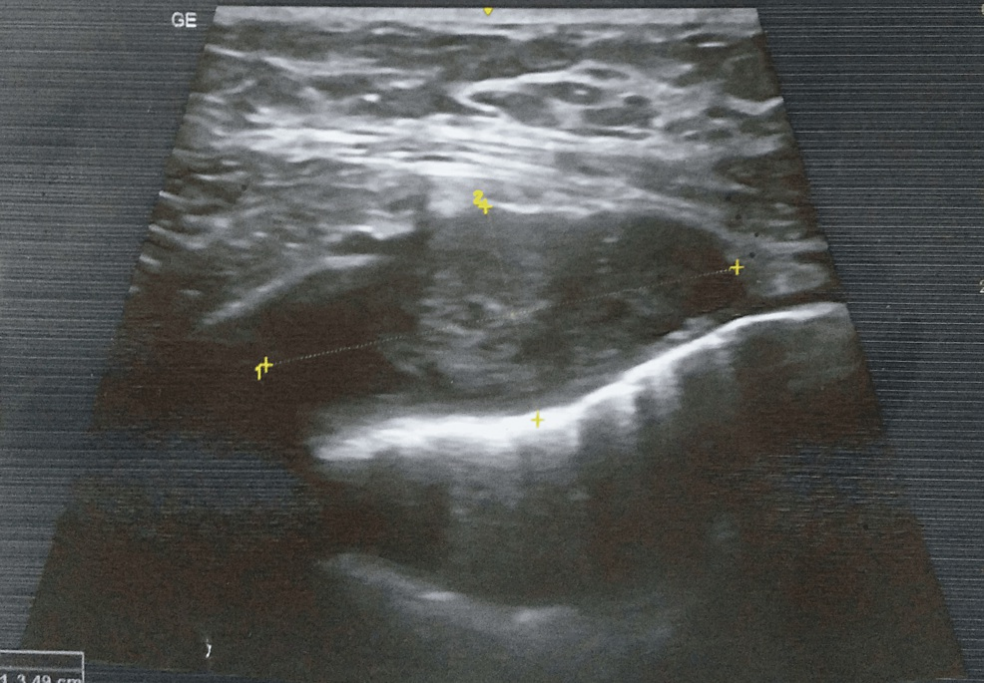
HRUSG of the right infraclavicular region was suggestive of intramuscular heterogenous collection HRUSG - high-resolution ultrasonography

**Figure 3:**
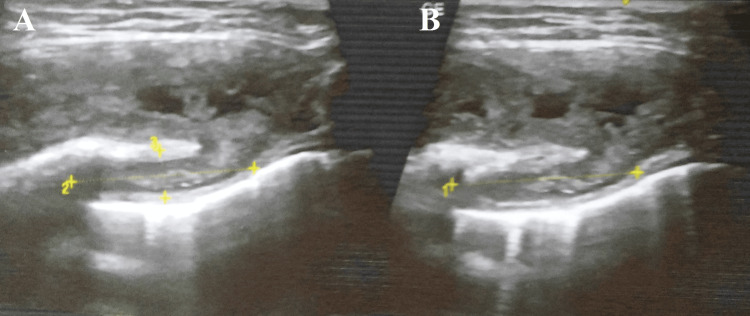
HRUSG showing a collection with deeper intrathoracic extension abutting and compressing anterior lung surface A: Collection with deeper intrathoracic extension. B: Collection showing deeper intrathoracic extension abutting and compressing anterior lung surface. HRUSG - high-resolution ultrasonography

A high-resolution ultrasonography of the right infraclavicular region was suggestive of an intramuscular heterogeneous collection of size 52x39x16 mm with a volume of approximately 16 cc with thick irregular walls in the right pectoralis muscle. This collection showed deeper intrathoracic extension, abutting and compressing the anterior lung surface. The intraathoracic component measured approximately 2.5x3.5x2 cm with a volume of 5 cc (Figures 2-3).

**Figure 4:**
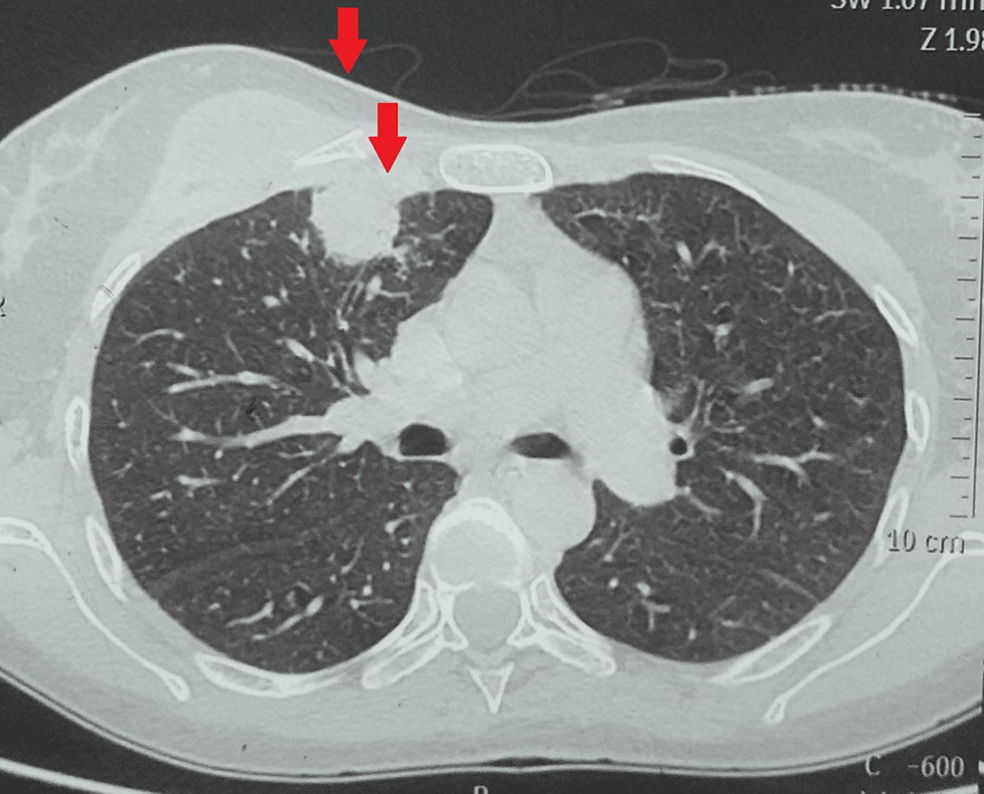
MDCT of the chest showing collection with moderately thick enhancing walls in the right pectoralis muscle MDCT - multidetector computed tomography

**Figure 5:**
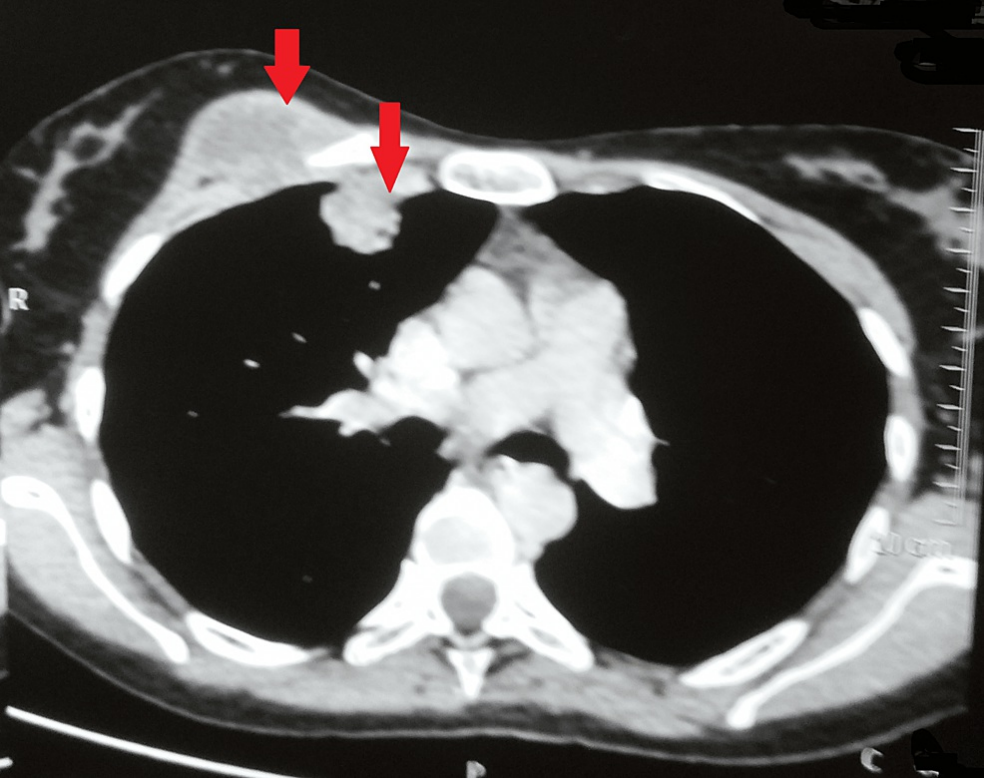
MDCT of the chest showing collection with moderately thick enhancing walls in the right pectoralis muscle MDCT - multidetector computed tomography

**Figure 6:**
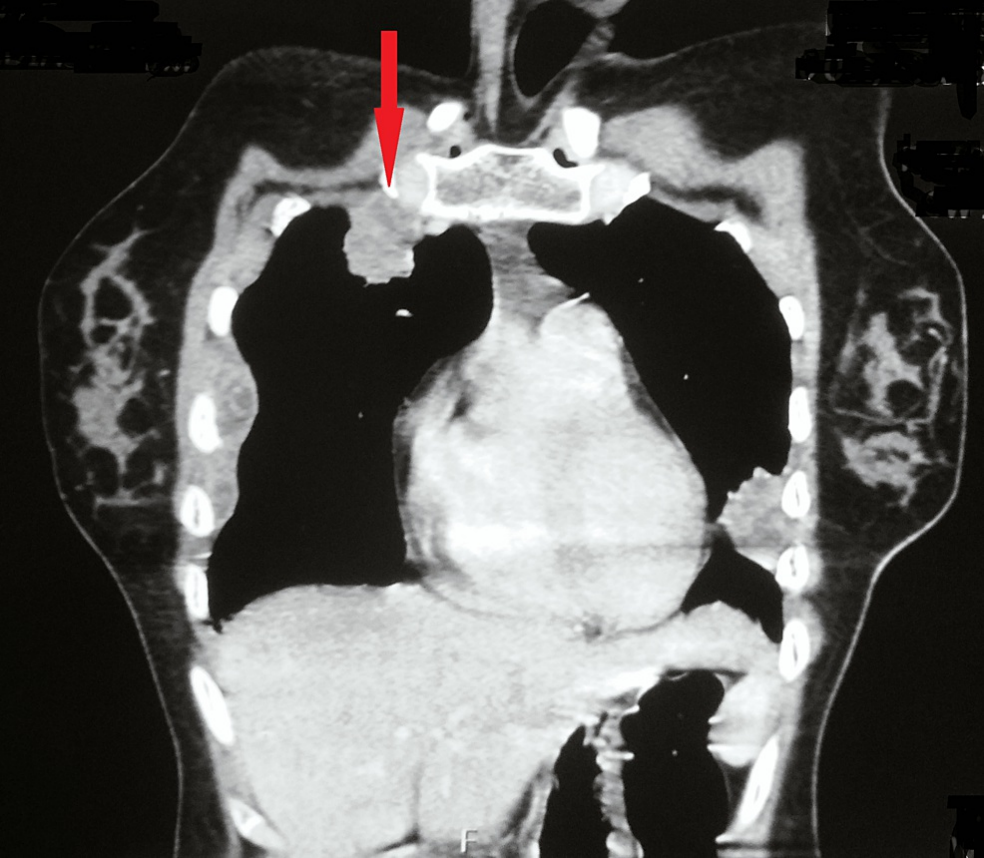
MDCT of the chest showing collection with moderately thick enhancing walls in the right pectoralis muscle MDCT - multidetector computed tomography

A multidetector computed tomography with the contrast of the chest showed a 45x27 mm well-defined collection with moderately thick enhancing walls in the right pectoralis muscle deep in the right breast. Another 29x27 mm pleural-based lobulated soft tissue density area with an eccentric pocket of the collection was seen in the right anterolateral chest wall in the first intercostal space. A few other similar morphology soft tissue density nodular lesions were scattered along the right and left, lateral, posterior, and medial chest walls. Some of these lesions showed small pockets of fluid. Atelactatic changes were seen in the underlying lung parenchyma, suggesting an infective etiology likely Koch's (Figures 4-6). 

Laboratory workup was remarkable for a raised erythrocyte sedimentation rate, i.e., 59 mm/hour, with a normal blood count. Her liver and renal function tests, along with anti-streptolysin O titers, were not deranged. An extensive laboratory workup, including antinuclear antibodies, antineutrophil cytoplasmic antibodies, C-reactive protein, thyroid function tests, tumor marker levels, and inflammatory markers, returned results within normal limits. The outcomes of several infection tests for syphilis, hepatitis (A, B, and C), and HIV (I and II) were negative.

A fine needle aspiration cytology was suggestive of degenerated acute on chronic inflammatory cells in the background of necrosis. On Ziehl-Neelsen staining, acid-fast bacilli were positive. The pus for a cartridge-based nucleic acid amplification test showed a detection of *Mycobacterium tuberculosis *(medium), and the same was confirmed with growth on an automated liquid culture Bactec™ Automated Blood Culture System (Becton, Dickinson, and Company, Franklin Lakes, NJ). Additionally, her induced sputum microscopy for acid-fast bacilli and cartridge-based nucleic acid amplification tests were negative.

An incision and drainage of the infraclavicular swelling were done with the removal of 15 ml of yellow-colored, non-fowl-smelling pus. She was initiated on antituberculous treatment with a fixed-dose combination of isoniazid, pyrazinamide, rifampicin, and ethambutol for 56 days, followed by isoniazid, rifampicin, and ethambutol for 112 days. A tablet of pyridoxine (10 mg) was added per the national guidelines. Presently, she has completed two months of her treatment with a significant reduction in her swelling and pain. Besides, there were no remarkable adverse drug reactions.

## Discussion

Extrapulmonary manifestations of tuberculosis are relatively infrequent and mainly seen in lymph nodes [1,8]. *Mycobacterium tuberculosis* can invade practically any area of the body, resulting in disease. Twenty to thirty percent of cases of extrapulmonary tuberculosis involve the musculoskeletal system, and the spinal bones continue to be the most common site of infection [9]. Further, musculoskeletal tuberculosis is commonly seen in immunocompromised patients and is infrequent in immunocompetent individuals like the present case.

Based only on the history and physical examination, the diagnosis of tuberculosis of the pectoralis muscles might be challenging because of the multitude of vague signs and symptoms. As a result, it calls for specialized investigations and a high degree of suspicion in the patients who report chest wall swellings, especially in the absence of constitutional signs and symptoms of tuberculosis [9]. However, this could cause a delay in the diagnosis and treatment.

Ultrasound-guided needle aspiration followed by smears or culture, as done in this case, is indicated to determine the underlying cause [9,10]. However, this procedure has its limitations, giving ambiguous results and having a 36.3% success rate for a definite diagnosis. Besides, the positive rate of culture was 60%, while the positive rate of acid-fast bacilli on smear microscopy is reported as 35%. Abid et al. reported surgical drainage and subsequent testing of the pus to establish the diagnosis as done in the present case [10].

Management is essentially medical, with antituberculous chemotherapy [11]. However, surgical drainage of the collection helps reduce the bacterial load, and the results are cosmetically better. An extensive review of the literature revealed that tuberculosis of the pectoralis with bilateral pleural base nodular lesions has never been reported in an immunocompetent female. A similar case was presented by Moyano-Bueno et al. in a 29-year-old Senegalese male [12]. However, the present case differs from theirs in gender, ethnicity, and the presence of bilateral multiple pleural base nodular lesions on computed tomography of the chest. A few other cases of tuberculosis of the pectoralis are mentioned in Table 1.

**Table 1:** Details of cases of tuberculosis of the pectoralis muscle

Case	Gender	Age in years	Areas of residence	Underlying disease and pulmonary lesions/history	Main clinical manifestations	Involved sites	Treatment duration
Morris et al. [13]	Male	9	Asia	Not any	Pain and swelling, which were tender and fluctuant	Pectoralis, forehead	Eight weeks
Winzer et al. [14]	Male	53	Europe	History of pulmonary tuberculosis	A fixed tender mass	Pectoralis	Chronic occult
Grigorakos et al. [7]	Male	24	Africa	Not any	Swelling and pain	Pectoralis	Two years
Grigorakos et al. [7]	Male	38	Africa	Not any	Swelling and pain	Pectoralis	Two years
Moyano-Bueno et al. [12]	Male	29	Senegal	Not any	Increase in size of the right hemithorax	Pectoralis major muscle	Not available
Present case	Female	26	India	Bilateral pleural base nodular lesions	Painful swelling	Pectoralis major muscle	On treatment for two months

Generally, the prognosis of tuberculosis of the pectoralis major is good, but Wang et al. reported that the mortality rate was 14.3% and was even higher, i.e., 30%, in patients with hematogenous tuberculous myositis [15]. In their study, Zeng et al. reported that two out of 16 patients died [16]. The patient reported here is doing fine and has completed two months of her treatment.

This particular case is significant because there is so little literature on these kinds of presentations. Nonetheless, this was but one case of this kind, which highlights the significance of extensive research from high-burden nations for precisely figuring out the course of illness, early detection, and prompt treatment.

## Conclusions

To conclude, a young Indian female with complaints of painful swelling over the right infraclavicular region was diagnosed with tuberculosis of the pectoralis muscle with fine needle aspiration cytology, a cartridge-based nucleic acid amplification test, automated liquid culture, and radiometric investigations. It requires a high index of suspicion to achieve such a rare diagnosis of muscular tuberculosis of the pectoralis muscle, as muscle tissue is presumed to be inert to bacterial infection. Besides, achieving this rare diagnosis was remarkable, especially in an immunocompetent female patient and in the absence of pulmonary involvement.
